# A prospective evaluation of treatment with Selective Internal Radiation Therapy (SIR-spheres) in patients with unresectable liver metastases from colorectal cancer previously treated with 5-FU based chemotherapy

**DOI:** 10.1186/1471-2407-5-132

**Published:** 2005-10-15

**Authors:** L Lim, P Gibbs, D Yip, JD Shapiro, R Dowling, D Smith, A Little, W Bailey, M Liechtenstein

**Affiliations:** 1 The Royal Melbourne Hospital, Parkville, Victoria, Australia; 2 Cabrini Hospital Malvern, Victoria, Australia; 3 The Canberra Hospital, Canberra, ACT, Australia

## Abstract

**Background:**

To prospectively evaluate the efficacy and safety of selective internal radiation (SIR) spheres in patients with inoperable liver metastases from colorectal cancer who have failed 5FU based chemotherapy.

**Methods:**

Patients were prospectively enrolled at three Australian centres. All patients had previously received 5-FU based chemotherapy for metastatic colorectal cancer. Patients were ECOG 0–2 and had liver dominant or liver only disease. Concurrent 5-FU was given at investigator discretion.

**Results:**

Thirty patients were treated between January 2002 and March 2004. As of July 2004 the median follow-up is 18.3 months. Median patient age was 61.7 years (range 36 – 77). Twenty-nine patients are evaluable for toxicity and response. There were 10 partial responses (33%), with the median duration of response being 8.3 months (range 2–18) and median time to progression of 5.3 mths. Response rates were lower (21%) and progression free survival shorter (3.9 mths) in patients that had received all standard chemotherapy options (n = 14). No responses were seen in patients with a poor performance status (n = 3) or extrahepatic disease (n = 6). Overall treatment related toxicity was acceptable, however significant late toxicity included 4 cases of gastric ulceration.

**Conclusion:**

In patients with metastatic colorectal cancer that have previously received treatment with 5-FU based chemotherapy, treatment with SIR-spheres has demonstrated encouraging activity. Further studies are required to better define the subsets of patients most likely to respond.

## Background

Colorectal cancer (CRC) is the most common GI malignancy accounting for 4718 deaths in Australia [1] and almost 437,000 deaths worldwide annually making it the most third most common malignancy in the developed world [2]. Around fifty to sixty percent of these patients will develop liver metastases, and in approximately 20% of cases the liver is the only site of disease at death[3]. Surgical resection of all apparent disease is possible in selected patients, however for the majority of patients with metastatic CRC the standard approach remains systemic chemotherapy.

Selective Internal Radiation (SIR) spheres (Sirtex Medical, Sydney, Australia) are a new radiotherapeutic treatment for liver metastases. These resin microspheres contain yttrium, a high energy beta-emitting isotope, and are embolised into the hepatic artery where they become lodged within the microvasculature of the tumour. The treatment is relatively selective as hepatic tumours derive their blood supply almost exclusively from the hepatic artery whereas normal liver parenchyma is supplied predominantly by the portal circulation. Animal studies suggest that SIR spheres allow on average 200–300 Gy to be delivered to liver tumours [4]. In contrast the delivery of standard external beam radiation therapy to the whole liver is limited by the ability of the normal parenchyma to tolerate only 30–35 Gy, an insufficient dose to produce a significant anti-tumour effect [5].

Encouraging results have been reported following previous studies of SIR spheres in metastatic colorectal cancer. In a series of 21 chemonaive patients with colorectal liver metastases who were randomised to receive intravenous 5FU alone or intravenous 5FU plus SIR spheres, the combination demonstrated a higher response rate and significantly improved progression free survival compared to chemotherapy alone [6]. In a larger study of 74 patients combining SIR-spheres with hepatic artery chemotherapy superior response rates and time to progression over treatment with chemotherapy alone were seen in patients with colorectal cancer [7]. With the exception of hepatocellular carcinoma[8] results in other tumour types have not been so encouraging [9].

We report here the first prospective series conducted to better define the efficacy and safety of SIR-spheres(Yttrium^90^) in patients with colorectal cancer and liver metastases that have previously received 5-FU based chemotherapy. No financial support was received from SIRTex for the purposes of this study.

## Methods

Data for consecutive patients with metastatic colorectal cancer treated with Sirtex microspheres were collected prospectively across 3 Australian centres from Jan 2002 and March 2004. During this period of the time both oxaliplatin and irinotecan were not reimbursable in Australia as part of first-line therapy for patients with metastatic colorectal cancer outside of a clinical trial. These agents were available for patients that had progressed following initial 5-FU based treatment.

Patients were informed of the available evidence regarding SIR-spheres treatment. Patients that elected to proceed with treatment were informed that data would be collected prospectively as part of a research project. Toxicities and protocols were outlined in accordance with the manufacturer's guidelines and were common across all participating centres. Patients were considered eligible if they had liver metastases from colorectal cancer with histological confirmation of their primary tumour. All patients were required to have measurable disease within the liver. Extra-hepatic disease was allowed if the liver was the dominant site of disease.

Patients with an expected survival of less than 3 months, documented brain metastases, or a poor performance status (ECOG >2) were excluded. Adequate hepatic, renal and liver function was required including a normal clotting profile, an albumin >30 g/L, bilirubin <20 umol/L and no evidence of liver decompensation such as ascites or portal hypertension was permitted. Patients with portal vein thrombosis were also excluded from this study.

Previous treatment with chemotherapy was allowed provided this had been more than 2 months prior to planned treatment with SIR-spheres. Patients received bolus 5FU chemotherapy concurrent with the SIR-spheres, as a radiosensitiser, and subsequently in responding patients at the investigator's discretion.

### Pre treatment workup and disease evaluation

All patients underwent a standard pre-treatment evaluation as per manufacturers guidelines. An hepatic angiogram was performed to define arterial anatomy prior to treatment and to permit a nuclear medicine scan with radio labeled Tc 99 m-MAA (macro-aggregated albumin) in order to exclude patients at high risk of lung (radiation pneumonitis) or GI toxicity (gastric/duodenal ulceration) due to hepato-systemic shunting or aberrant vasculature. The fraction of extra-hepatic shunting was determined for each patient as a percentage. Patients who had shunting of greater than 20% were excluded from the study. Shunting of between 12 and 20% resulted in a reduction in the dosage of spheres delivered. The final dose of SIR-spheres administered in units of GBq was calculated according to each patient's body surface area and percentage of tumour involvement of liver (an assessment made by the radiologist after viewing the baseline CT scan). Across the three participating centres, the workup and delivery of SIR spheres was performed by a total of four experienced interventional radiologists with two centres having all treatment administered by a single interventional radiologist.

Following a single treatment of SIR-spheres, two weeks after the MAA scan, patients were routinely followed and assessed at monthly intervals. Acute toxicity was assessed initially and at subsequent clinical visits. All toxicities were graded according to National Cancer Institute (NCI) Common Toxicity Criteria, and performance status according to Eastern Cooperative Oncology Group (ECOG) criteria. Disease evaluation was performed by CT imaging at 2 months and bi-monthly thereafter until disease progression. A complete response (CR) was defined as the disappearance of all target lesions. A partial response (PR) was defined as >30 percent decrease in the sum of the longest dimension of the target lesions at 2 months. All responses were confirmed on repeat imaging. Progressive disease (PD) was defined as a >20 percent increase in the sum of the longest recorded target lesion(s) within the liver or the development of new or progressive extrahepatic disease. Stable disease (SD) was defined as a decrease not sufficient to qualify for a PR nor an increase not sufficient to qualify for PD. The results of all staging investigations and toxicity assessments were collected and analysed prospectively. The duration of response was determined from the date of the first response evaluation at 2 months until progressive disease.

## Results

### Patient characteristics

A total of 30 patients underwent treatment between January 2002 and March 2004.(see table 1). There were 22 males and 8 females with a median age of 61.7 years (range 36–77). Ninety percent of patients had an ECOG performance status of 0 or 1. Twenty percent of patients (6) had low-volume extra-hepatic disease, the remainder of the patients had liver-only disease. All patients had failed 5FU chemotherapy and 22 (73%) patients had failed either oxaliplatin or irinotecan containing regimens, with 14 (46%) having progressed through both. 21 of the 30 patients received 5-FU concurrent with the SIR-spheres.

Of the 30 patients treated, 29 are evaluable for disease response. One patient presented shortly after treatment with worsening liver function tests, liver failure and death considered by the investigator to likely be from rapid disease progression. This was not confirmed with imaging. Overall, there were 10 partial responses (33% of all 30 patients treated). Of note ongoing responses were seen in a number of patients, with continued reduction in the size of liver lesions occurring out to 12 months from treatment. One patient with an initial partial response had achieved a complete response at six months. The median duration of response is 8.3 months (range between 2–18+ months) at a median follow up time (as of July 2004) of 18.3 months for all patients. Eight patients in total had stable disease at 2 months (27%) and the remaining 12 (40%) had disease progression (n = 11) or were not evaluable (n = 1). The median time to progression for all patients was 5.3 mths, however, patients who achieved a partial response within the liver as a group had a median progression free survival of 9.2 mths. See table 2

All responses occurred in patients with disease confined to the liver (n = 24) and no responses were seen in patients with a performance status of 2 (n = 3). In the 8 patients that had received only prior 5-FU there were 6 responses (75%). In the patients that had received oxaliplatin and/or irinotecan (n = 22) there were 5 responses (23%). This included responses in 3 of 14 (21%) patients that had previously received all standard chemotherapy options. No other factors were apparently predictive of response, including the bulk of disease, in this study.

### Toxicity

Overall toxicity assessments were carried out according to NCI common toxicity criteria at all three centres. The findings were consistent across all study sites with between 2–8 weeks of lethargy, anorexia, nausea and RUQ pain being observed to a variable extent in most patients. However, in most cases, these side effects were mild and self-limiting following treatment with standard anti-emetics and analgesics medication. Three patients reported severe nausea and lethargy for 2 weeks following treatment and moderate symptoms were reported in several patients up to 1 month following treatment.

**Figure 3:**
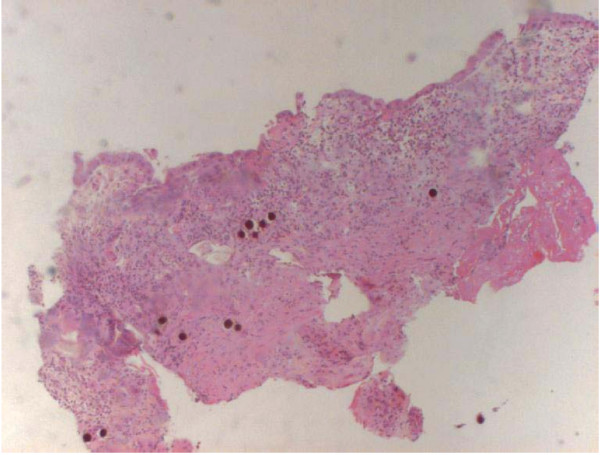
Biopsy of gastric mucosa showing inflammation (gastritis) from several SIR spheres, clearly visible.

Serious treatment related toxicities clearly related to treatment with SIR-spheres were recorded in 4 patients (13%) who had gastric/duodenal ulceration confirmed on gastroscopy. In 3 of these patients, SIR-spheres were seen in biopsies taken from ulcerated mucosa (Figure 3) and in two patients there was rapid improvement following treatment with proton pump inhibitors. However, one patient had severe ongoing and disabling pain, anorexia and nausea that continued until the time of death 3 months later and another patient experienced ongoing symptoms beyond 3 months, despite the use of proton pump inhibitors.

Other serious toxicity potentially related to SIR-spheres included a single patient who was admitted to hospital with acute right upper quadrant pain and marked deterioration in her liver function tests one month following treatment with SIR-spheres. The clinical assessment was that the patient likely had radiation hepatitis. Her symptoms settled with conservative management.

## Discussion

This prospective evaluation documents the experience in our three institutions of using Selective Internal Radiation spheres to treat liver metastases from colorectal cancer in patients that had previously received 5-FU chemotherapy. Our series adds to the published experience documenting the activity of this treatment in selected patients, along with the potential for significant toxicity. For the period of this study oxaliplatin and irinotecan were available in Australia only for patients that had progressed following initial 5-FU based treatment and this is reflected in the study design.

Overall in our experience, treatment with SIR-spheres demonstrated promising activity in pre-treated patients with liver metastases from colorectal cancer. Partial responses were seen in six of the eight patients that had failed 5FU alone. This includes one patient that was able to undergo potentially curative resection of residual liver disease after further response to systemic chemotherapy and she remains disease free 22 months later. The response rate in our series is similar to that reported in the previous small randomized study where this combination was used first-line [7]. The progression free and overall survival data for our study are shown in Figure 3. Due to the small patients numbers this activity may partly reflect chance or patient selection. However, this is encouraging efficacy as the alternative treatment for these patients would have been irinotecan alone or oxaliplatin plus 5-FU where responses are typically seen in less than 20% of patients [10,11].

Significant response rates were also seen in patients that had progressed through several lines of chemotherapy. Notably, fourteen of these patients had previously received both irinotecan and oxaliplatin, and the response rate was maintained in this group. The only treatment other option open to these patients would be cetuximab, a monoclonal antibody directed at the epidermal growth factor receptor (EGFR). As a single agent responses are seen in about 10% of patients [12] and response rates are higher when concurrent irinotecan is administered [12]. However, not all patients are suitable for cetuximab as approximately 25% of patients do not express the EGFR[13]. Based on our results SIR-spheres appear to be a good option for patients with colorectal cancer who have liver only disease and maintain a good performance status following progression on all standard chemotherapy drugs. The suggestion from our results is that benefit from the addition of SIR-spheres will be greater if used earlier, but this will need to be confirmed in larger studies.

The toxicity data from our experience was consistent with findings from earlier trials involving SIR-spheres. The 13% severe gastric/duodenal ulceration rate is significant and is consistent with a recent larger study reporting a 12% GI ulceration rate [14]. Gastrointestinal ulceration occurred despite strictly adhered to protocols of pre-treatment workup and treatment only by experienced interventional radiologists. The product information recommends routine use of a H-2 antagonist prophylactically the day before the procedure and for a month afterwards in view of the known association of peptic ulceration with SIR-spheres treatment [15]. Although this was not part of our treatment protocol, the prophylactic use of proton-pump inhibitors or H-2 antagonists should be considered in patients treated with SIR-spheres. In our analysis there were no apparent indicators of which patients were likely to experience toxicity. In particular this did not appear related to disease bulk or patient performance status.

## Conclusion

In summary, this series has demonstrated that treatment with SIR-spheres produces encouraging responses in pretreated patients with colorectal cancer. Further studies in this group of patients should be pursued. On the basis of our results, thought should be given to stratifying patients according to the presence or absence of extrahepatic disease and according to performance status. We have also demonstrated the potential for significant toxicity, and such treatment should only be conducted in centres with experienced interventional radiologists.

The results of ongoing phase I/II trials that are exploring the combination of SIR-spheres with multi-agent chemotherapy (including irinotecan and oxaliplatin containing regimens) are eagerly awaited. Ultimately however, there remains a need for large phase III clinical trials evaluating the efficacy of standard therapy plus SIR-spheres compared to standard therapy alone. Such trials will define the true value of SIR-spheres and will permit insight into optimal patient selection. Finally the financial costs of this new treatment are not insignificant and future studies will also need to clearly demonstrate cost effectiveness as well as efficacy in an environment where the costs of treating patients with colorectal cancer are rapidly escalating [16].

## Pre-publication history

The pre-publication history for this paper can be accessed here:



## Figures and Tables

**Figure 1:**
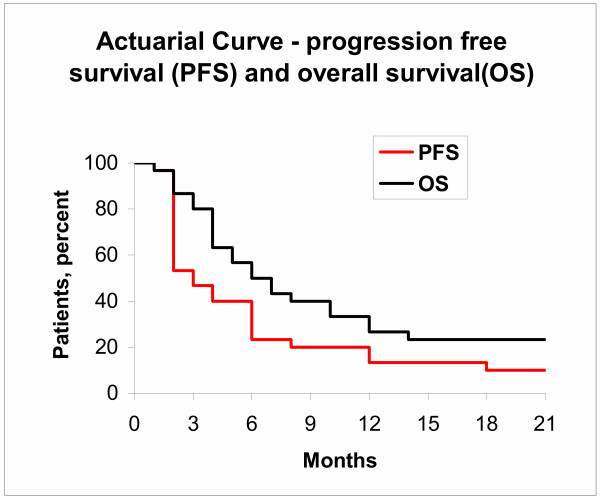
Survival curves for the 30 patients treated.

**Figure 2:**
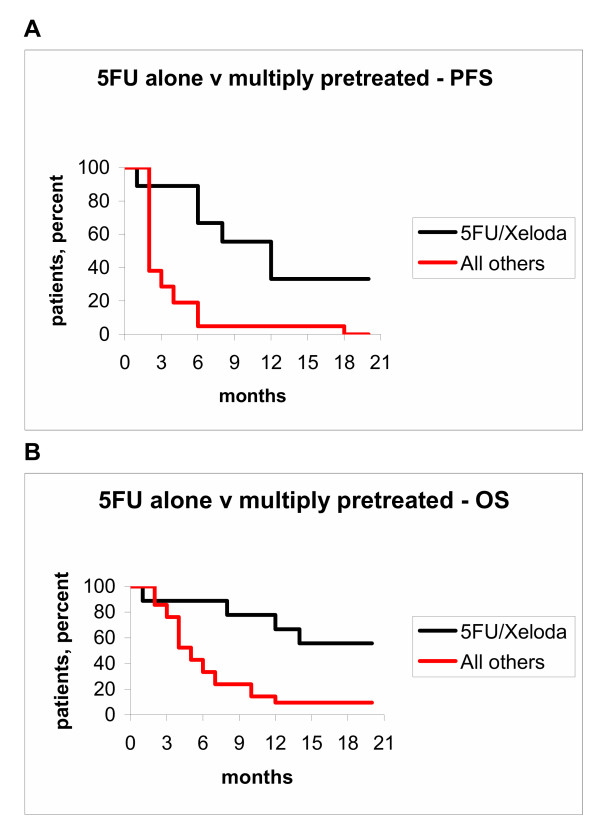
(A) Progression free survival for the two groups of patients, those that had previously received 5-FU or xeloda, and those that had received at least one oxaliplatin or irinotecan containing regimen. (B) Overall survival for the two groups of patients, those that had previously received 5-FU or xeloda, and those that had received at least one oxaliplatin or irinotecan containing regimen.

**Table 1:** Patient Characteristics

Total Number	30
Male	22 (73%)
Female	8 (27%)
ECOG 0	16 (53%)
ECOG 1	11 (37%)
ECOG 2	3 (10%)
Extra-hepatic disease	7 (20%)
Failed 5FU alone	8 (27%)
Failed 5FU + CPT-11 **and **Oxaliplatin	14 (46%)
Failed 5FU + **either **CPT-11 **or **Oxaliplatin	8 (27%)

**Table 2:** Results At 2 months by patient group (*1 patient died prior to the first evaluation and has been classified as progressive disease).

	Partial Response (%)	Stable Disease (%)	Progressive Disease (%)
Overall (n = 30)	10 (33%)	8 (27%)	12 (40%)*
Failed 5FU alone (n = 8)	6	1	1*
Failed 5FU and CPT-11 **and **Oxal (n = 14) containing regimens	3	2	8
Failed 5FU and **either **CPT-11 **or **Oxaliplatin (n = 8) containing regimens	2	2	4
Extra-Hepatic disease (n = 7)	0	2	5
ECOG 2 (n = 3)	0	1	2
